# Gut Microbiota Mediates Protection Against Enteropathy Induced by Indomethacin

**DOI:** 10.1038/srep40317

**Published:** 2017-01-09

**Authors:** Xue Xiao, Geicho Nakatsu, Ye Jin, Sunny Wong, Jun Yu, James Y. W. Lau

**Affiliations:** 1 Department of Gastroenterology, West China Hospital, Sichuan University, Chengdu, China; 2 Institute of Digestive Disease and Department of Medicine and Therapeutics, State Key Laboratory of Digestive Disease, Li Ka Shing Institute of Health Sciences, The Chinese University of Hong Kong, Hong Kong; 3 Department of Surgery, The Chinese University of Hong Kong, Hong Kong

## Abstract

Non-steroidal anti-inflammatory drugs (NSAIDs) can cause significant small bowel injuries. The role of gut microbiota in this NSAID-induced enteropathy is poorly understood. We studied the dynamic changes in gut microbiota following indomethacin administration in mice, and investigated the effects of these adaptive changes on subsequent NSAID-induced enteropathy. The changes in gut microbiota were studied using 16S rRNA sequencing, and the effects of such changes were investigated using antibiotics and a faecal transplantation model. After indomethacin treatment, significant adaptive changes in gut microbiota were observed, including increased abundance of Firmicutes and decreased abundance in that of Bacteroidetes. Depletion of gut microbiota with antibiotics led to a higher mortality (*P* = 0.0021) in mice compared to controls. Mice pre-transplanted with adaptively changed microbiota showed less small bowel injury and lower levels of pro-inflammatory cytokines when exposed to indomethacin. In summary, this study identifies adaptive changes in the gut microbiota upon indomethacin administration, which can in turn ameliorate further NSAID-induced injury. The heightened mortality with antibiotic depletion of the adaptively changed microbiota suggests its important role in protecting against such injury. This study provides insight for future efforts to target the microbiota as a therapeutic strategy.

Non-steroidal anti-inflammatory drugs (NSAIDs) are extensively used in clinical practice for their anti-pyretic, anti-inflammatory and analgesic properties. They however can cause small bowel injuries complicated by bleeding, perforation and stenosis. Visible damages or bleeding on small bowel are observed in up to 70% of chronic NSAID users^1^. Nevertheless, there is currently no proven-effective therapy for treatment of NSAID-induced small bowel injury other than drug withdrawal^2^,^3^.

The pathogenesis of NSAID-induced enteropathy is complex. NSAIDs suppress prostaglandin synthesis and impair the mucosal defense in small bowel. The enterohepatic re-circulation of NSAIDs further enhances the direct cytotoxic action on enterocytes^3^. Commensal microbiota has been implicated in the pathogenesis of NSAIDs enteropathy. Germ-free mice are resistant to indomethacin-induced small bowel injuries, but would become susceptible when colonized with commensal microbiota^4^,^5^. The use of antibiotics has also been shown to reduce indomethacin-induced small bowel injuries in rats^6^,^7^. Gut commensal microbiota has been shown to change rapidly after NSAID administration in animal models^8^,^9^. The effects of these adaptive changes in gut microbiota in response to NSAIDs is not clear. In our experiments, we studied how the dynamic changed gut microbiota could influence pathogenesis of indomethacin-induced enteropathy.

## Results

### Antibiotic depletion of gut microbiota resulted in an increased mortality in indomethacin treated mice

**Figure 1:**
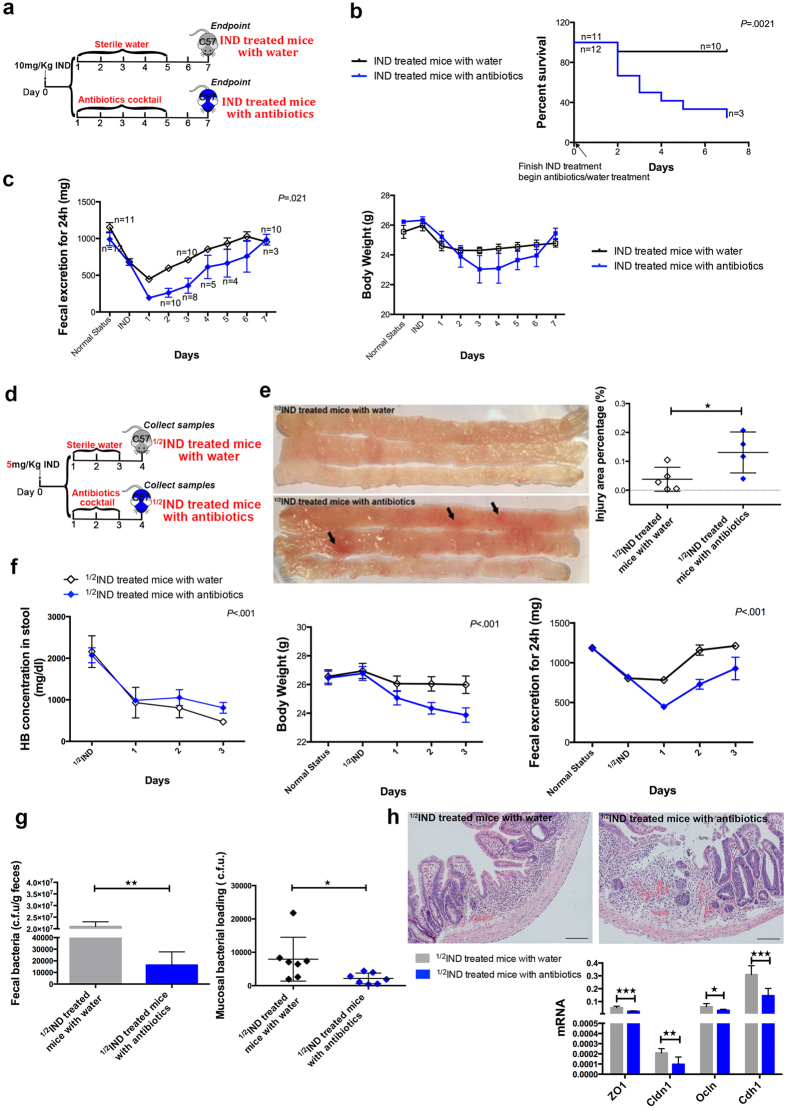
Eradication of gut microbiota in indomethacin treated mice caused a worse prognosis. (**a**) Experimental scheme shows after indomethacin treated for 24 hours, antibiotic cocktail or sterile water was administered (IND, indomethacin); (**b**) The survival rate was recorded daily, and analyzed by Kaplan-Meier survival analysis, every number besides the dot means the survived mice number; (**c**) Faecal weight (Left) and body weight (right) were recorded daily also, every dot represents the mean faecal weight or body weight of survived mice, error bars represent SEM, repeated two-way ANOVA was used; (**d**) In the experimental design, samples were collected 24 h after 3-day course of antibiotic cocktail or sterile water treatment, ^1/2^IND means 5 mg/kg indomethacin; (**e**) Representative pictures for injury area (arrows, 5 mice in each group at beginning, one in antibiotics treated group died on Day 2), and injury area percentage data, every dot represents one mouse; (**f**) Every dot represents the mean of stool haemoglobin concentration (right), body weight (middle) and faecal weight (left) of each group, the error bars represent SEM, repeated two-way ANOVA was used, n = 7 for each group; (**g**) PCR-based quantification of total faecal bacteria burden and mucosal bacteria burden, every dot represents one mouse, C.F.U, colony forming units; (**h**) H&E pictures show the histological changes during recovery, the bottom graph shows real-time PCR analysis of tight junction genes in the small bowel (*ZO1*, tight junction protein 1; *Cldn1*, claudin 1; *Ocln*, occludin; *Cdh1*, cadherin), n = 7 for each group. ^★^*P* ≤ 0.05, ^★★^*P* ≤ 0.01, ^★★★^*P* ≤ 0.001.

We first studied effects of the native gut microbiota on indomethacin-induced injury in mice. After a single treatment of 10 mg/kg indomethacin, mice were administered with antibiotics or sterile water (as control) for 5 days. The mice were observed for body weight changes, gastrointestinal function as indicated by daily faecal excretion, and also the survival rate (Fig. 1a). Despite this normally sub-lethal dosage of indomethacin with only 1 out of 11 mice died, a significantly increased mortality was observed in the antibiotics group with 8 out of 12 mice died (Fig. 1b, *P* = 0.0021 from Kaplan-Meier survival analysis). Post-mortem examination showed that mice died in antibiotics group suffered from severe peritonitis and small intestinal perforation, where the only deceased mouse in the control group died from small intestinal bleeding (Supplementary Fig. S1). Moreover, after depletion of gut microbiota with antibiotics, mice showed less daily faecal excretion as well as more macroscopic injury of their small bowel at the endpoint (Fig. 1c, Supplementary Fig. S2).

Given the high mortality in the antibiotics group, we next halved the indomethacin dosage to allow observation of the patho-physiological process upon recovery (Fig. 1d). Still, one mouse in antibiotics treated group died. We observed a poorer healing process in mice given antibiotics, which had a significantly higher haemoglobin concentration in stool (*P* < 0.001), lower body weight (*P* < 0.001), decreased faecal excretion (*P* < 0.001), and larger injury area percentage of small bowel (0.1306 ± 0.0354% vs 0.0379 ± 0.0186%, *P* = 0.043) (Fig. 1e,f). To confirm depletion of gut microbiota, we studied bacterial loads in both small bowel tissues and faeces and found significant reduction after antibiotics when compared to controls (Fig. 1g). The mucosal integrity was worse in the antibiotics treated mice than control mice with lower expressions of genes (*ZO1, Cldn1, Ocln, Cdh1*) involved in epithelial tight junctions (Fig. 1h). These gene expressions were confirmed to be reduced according to the severity of indomethacin-induced injury (Supplementary Fig. S3). The inflammatory response was further determined between groups, and were found not to be exacerbated in the antibiotics treated mice (Supplementary Fig. S4).

To eliminate the possibility that antibiotics used in the above experiments was toxic for mice and caused the above changes other than gut microbiota. We treated mice firstly with antibiotics, and then indomethacin was given (Supplementary Fig. S5). Results were consistent with published data, showed that by pretreating the same antibiotics, mice were protected from indomethacin with a lower haemoglobin concentration in stool and injury area percentage in small bowel than the control ones. Therefore, the same antibiotics, by eradicating gut microbiota, made different effects on mice when given before or after indomethacin may indicate that the role of gut microbiota was changed after indomethacin treatment.

### Indomethacin treatment induced adaptive changes in gut microbiota

**Figure 2:**
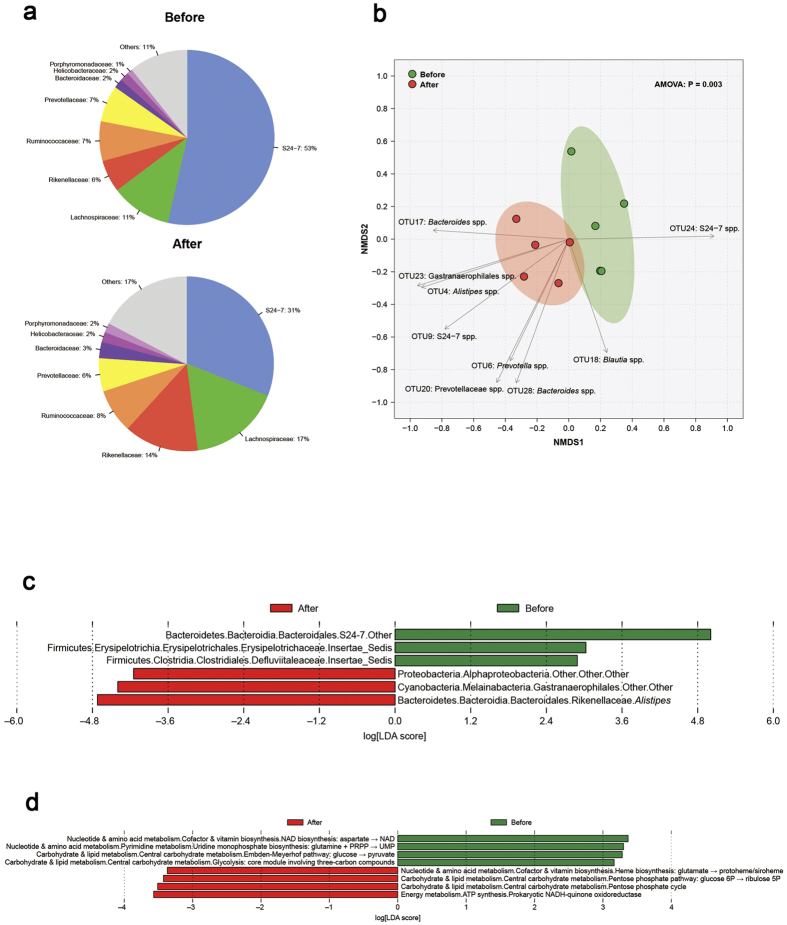
Indomethacin treatment induced adaptive changes in gut microbiota. 16S rRNA sequencing results are shown. (**a**) is Pie chart for faecal bacterial composition (family level); (**b**) is the result of Principle component analysis, every dot represents one sample, analysis of molecular variance (AMOVA) was used. Linear discriminant analysis data for (**c**) faecal bacterial changes and (**d**) their functional characterization are also shown.

We studied the gut microbial changes after indomethacin treatment. After 2 days of indomethacin treatment, we observed a distinct change in gut microbiota (*P* = 0.003 from principal component analysis) (Fig. 2a,b), characterized by a decreased proportion of Bacteroidetes (69 to 56%, including Porphyrominadaceae, Bacteroidaceae, Prevotellaceae, Rikenellaceae, S24-7) and an increased proportion of Firmicutes (18 to 25%, including Ruminoccaceae and Lachnospiraceae). With linear discriminant analysis, the abundance of Alphaproteobacteria, Gastranaerophilales and *Alistipes* was significantly higher after indomethacin treatment, whereas the abundance of S24-7, Erysipelotrichaceae and Defluviitaleaceae was significantly lower (Fig. 2c). We next grouped genes according to KEGG (Kyoto Encyclopedia of Genes and Genomes) modules. After indomethacin treatment, the altered microbiota were different in function when compared to the commensal ones. The altered microbiota were predominately involved in ATP and heme syntheses, and pentose phosphate metabolism (Fig. 2d).

**Figure 3:**
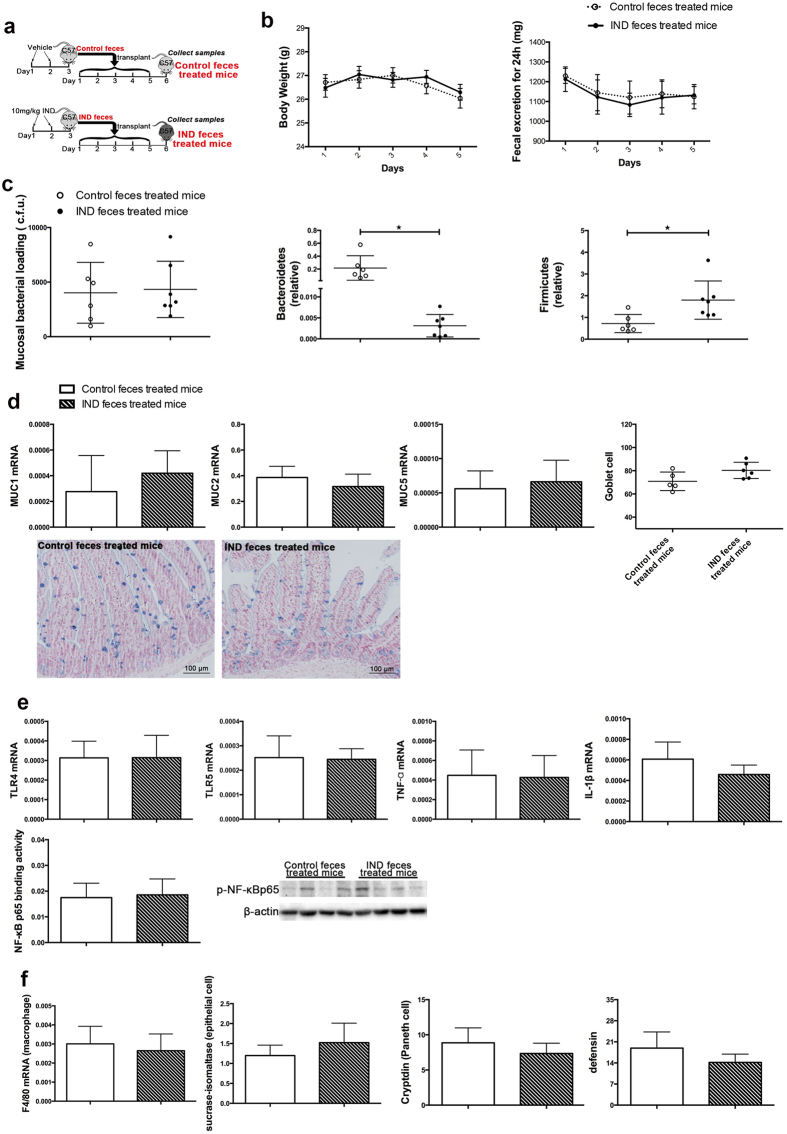
Indomethacin induced microbial alteration did not damage the normal small bowel mucosae. (**a**) Experimental scheme; IND, indomethacin; (**b**) Body weight and 24 h faecal weight records are shown as mean and SEM; (**c**) PCR-based quantification of total bacterial burden and relative levels of Bacteroidetes and Firmicutes 16S rRNA in small bowel mucosae, every dot represents one mouse, C.F.U, colony forming units; (**d**) The mucus barrier was assessed by mRNA levels of MUC1, MUC2, MUC5, and goblet cells were stained with Alcian blue solution and quantified, every dot represents one mouse, mean and SD are shown; (**e**) Bacterial receptors as TLR4 and TLR5 and inflammatory cytokines including TNF-α, IL-1β were detected with real-time PCR, NF-κB p65 binding activity and Protein level were checked by ELISA and Western blot with nuclear protein; (**f**) The immune response factors as macrophage, epithelial cells, Paneth cell, and defensin were detected with differential markers by real-time PCR. n = 3–6 for control faeces treated mice, n = 6–8 for IND faeces treated mice, mean and SD are shown. Student’s *t* test was used.

We further used a faecal transplantation model to understand effect of the gut microbiota after indomethacin treatment on intestinal tract. We treated the donor mice with 2 days of indomethacin at 10 mg/kg/day (Supplementary Table S1, Supplementary Fig. S6), and collected stool from these indomethacin-treated mice or controls. The stools were then fed to recipient mice for 5 days (Fig. 3a). We measured and found similar daily body weight and faecal excretion in either group (Fig. 3b). Despite a decreased ratio of Bacteroidetes and increased ratio of Firmicutes in the small bowel tissue of the recipient mice (Fig. 3c) indicating an effective faecal transplantation, no phenotypic or molecular difference was observed between the two groups. There was no mucosal injury observed in the stomach, small bowel or colon in either group of the mice. There was no difference in the expression of mucin genes *MUC1, MUC2* and *MUC5*, or the number of mucus-producing goblet cells between the two groups (Fig. 3d). Consistently, the expression of factors involved in the recognition of bacteria (TLR4, TLR5), and immune response to bacteria (TNF-α, IL-1β, NF-κΒ and differential markers of macrophage, epithelial cell, Paneth cell and antimicrobial peptide) showed no significant changes in the small bowel of mice after being transplanted with the adaptively changed microbiota (Fig. 3e,f).

### Adaptive changes in gut microbiota protect mice against subsequent indomethacin-induced small bowel injury

**Figure 4:**
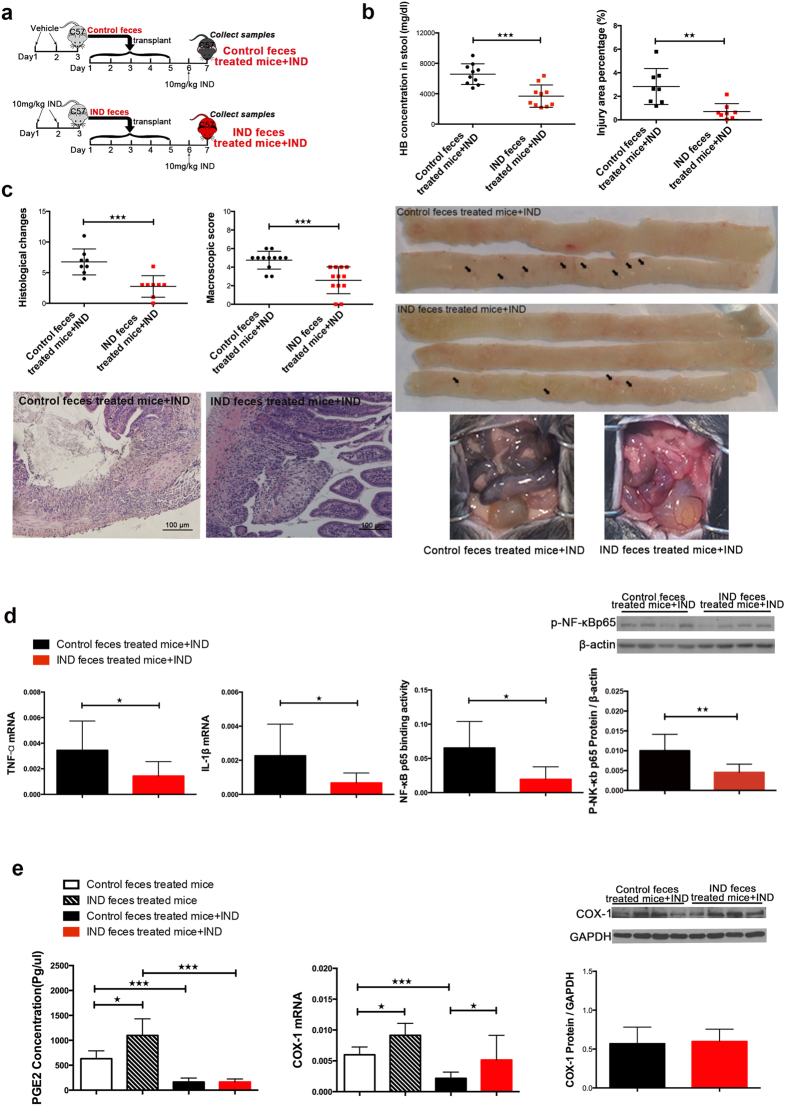
Adaptive changes in gut microbiota protect mice against indomethacin-induced small bowel injury. (**a**) Experimental scheme, all mice would be sacrificed 24 hours after indomethacin administration; Injury indices includes (**b**) stool haemoglobin concentration, injury area percentage (with pictures shown below the graph, arrows show injury lesions), and (**c**) histological changes (H&E staining picture shown below the graph), clinical assessment of small bowel injury (representative pictures shown in the lower right corner), every dot represents one mouse; (**d**) Inflammation examination, expression of IL-1β and TNF-α was detected with real-time PCR, NF-κB p65 binding activity was examined by ELISA, and nuclear protein was extreacted from small bowel tissue for western blot with anti-phosphate-NF-κB p65 antibody, mean and SD are shown; (**e**) Small intestinal PGE2 concentration was detected with ELISA, and COX-1 was detected with western blot and real-time PCR, mean and SD are shown. n = 6–8/group. Student’s *t* test was used, ^★^*P* ≤ 0.05, ^★★^*P* ≤ 0.01, ^★★★^*P* ≤ 0.001.

Mice transplanted with either faeces from indomethacin treated mice or controls were then administered with oral indomethacin 10 mg/kg (Fig. 4a). After 24 hours, mice transplanted with adaptively changed microbiota showed significantly reduced injury indices, including stool haemoglobin concentration (3686.32 ± 464.77 mg/dl vs 6576.19 ± 429.05 mg/dl, *P* < 0.001), injury area percentage (0.70 ± 0.24% vs 2.83 ± 0.54%, *P* = 0.003), histological score (2.75 ± 0.62 vs 6.75 ± 0.75, *P* = 0.001) and macroscopic score (2.58 ± 0.42 vs 4.75 ± 0.28, *P* < 0.001), with decreased expressions of pro-inflammatory cytokines, such as TNF-α, IL-1β, and lower NF-κB p65 activity (Fig. 4b–d). After indomethacin treatment, COX-1 and PGE2 were similarly suppressed in both groups, suggesting that the pharmacodynamics of indomethacin have not been disturbed by the faecal transplantation (Fig. 4e).

**Figure 5:**
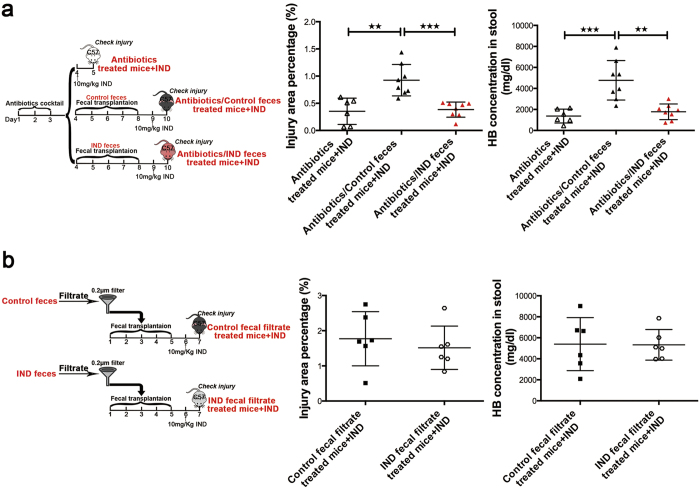
Microbiota from indomethacin treated mice was protective. (**a**) Gut microbiota depletion model: mice were pre-treated with 3-day course of antibiotics, faecal transplantation and indomethacin were then given as before; (**b**) “sterile faeces” faecal transplantation model: bacteria were filtered with a 0.2 μm filter, then faecal transplantation and indomethacin were given. The small intestinal injury area percentage data and stool haemoglobin concentration data were shown according to experimental schemes in the left part, every dot represents one mouse, mean and SD are shown, Student’s *t* test was used, ^★★^*P* ≤ 0.01, ^★★★^*P* ≤ 0.001.

A similar protective effect of this transplanted microbiota was observed, when these mice were pre-treated with 3-day course of antibiotics to deplete the microbiota. After antibiotics pretreatment, faecal transplantation and indomethacin were given as before (Fig. 5a). We observed significant small bowel protection after transplantation with adaptively changed microbiota when compared to transplantation with normal commensal microbiota (Injury area percentage 0.38 ± 0.05% vs 0.92 ± 0.10%, *P* < 0.001; stool haemoglobin concentration 1775.37 ± 744.66 mg/dl vs 4773.68 ± 1881.90 mg/dl, *P* < 0.001). These data further support the protective role of the changed microbiota.

We further filtered bacteria from faecal suspension with a 0.2 μm filter, and investigated effect of the filtered faeces on subsequent indomethacin-induced small bowel injury (Fig. 5b). No difference in the injury area percentage and stool haemoglobin concentration was found in either group of mice treated with the filtrates. This further supports that the protective effect was likely to come from the adaptively changed microbiota, rather than other soluble filtrates from the faeces.

## Discussion

The role of gut microbiota in NSAID induced enteropathy has not been fully defined. In germ free mice and antibiotics-depleted mice, small bowel injury is much attenuated following NSAID administration^4^,^5^,^6^. These studies suggest that gut microbiota as a whole is requisite to small bowel injury. Other studies, however, suggest that certain members of the microbiota are protective^5^,^10^. Lactobacillus and Bifidobacterium can repress the growth of gram-negative bacteria which are increased in rats with ileal ulcers. These bacteria include *Eubacterium limosum* and *Escherichia coli* are thought to be ulcerogenic^5^. Our series of experiments confirmed that the administration of NSAID is deleterious to the small bowel of mice in the presence of commensal gut microbiota. We also learnt of the alteration in gut microbiota after administration of NSAID, and showed in a FMT (faecal microbiota transplantation) model that faeces harvested from mice with prior NSAID exposure are protective. Our findings suggest that gut microbiota can adapt to protect mice against the pathogenic process.

In our study, several of the bacterial taxa were shown to change following indomethacin administration. These altered taxa, as suggested by the KEGG modules, were involved in metabolism pathways on lipid synthesis, haemoglobin production and cell growth^11^,^12^. Their functions were distinct from those of the commensal ones. These adaptive changes also indicated an increase in the abundance of gram-positve bacteria families, including Lachnospiraceae. Butyric acid is a short-chain fatty acid, produced by several Lachnospiraceae, and could help to maintain the intestinal mucosal integrity^13^. On the other side, the decreased abundance in gram-negative bacteria families could also contribute to the protective effects of adaptively changed microbiota, as gram-negative bacteria may exacerbate the injury by activating TLR4 related inflammation^7^. Though a previous study showed a different gut microbial alteration after indomethacin treatment in mice^14^, the present study differs by employing 16S rRNA sequencing on serial samples from the same mouse, reducing individual variations to visualize the gut microbial alteration. And these compositional changes in microbiota are consistent with another sequencing study^15^.

Faecal transplantation is an efficient way to engraft the microbiota and transfer the phenotype of donors^16^,^17^,^18^,^19^, as shown in our study. We find that adaptive changes in gut microbiota do not exacerbate the damages but rather protect the mucosae from indomethacin-induced injury. Many previous studies showed that pretreating animals with single antibiotic, as metronidazole^20^,^21^, or antibiotic cocktail including neomycin, polymixin and bacitracin^6^, protected mice from NSAIDs enteropathy by depleting commensal microbiota. This observation is further confirmed in our study by pretreating mice with an antibiotic cocktail. However, the same antibiotic cocktail used in our study, when given after indomethacin results in a worse survival in mice, These discrepant outcomes are consistent with our sequencing data, which predicts disparate physiological effects of adaptively changed microbiota after indomethacin treatment. Pre-treatment with antibiotics eradicate the commensal microbiota, and ameliorate injuries in gut mucosae. On the contrary, antibiotics administered after indomethacin treatment exacerbates the injuries by eradicating the adapted bacteria. Some bacteria are protective whereas some are harmful and ulcerogenic^22^,^23^. These data suggest divergent roles of gut microbiota before and after indomethacin treatment, and signify that compositional difference in microbiota may arise after different antibiotic choice and timing to result in varying clinical phenotypes.

Antibiotics alone, such as penicillin G, erythromycin and metronidazole, can induce a low grade inflammation in gut after a long time treatment^24^,^25^. In our experiments, antibiotics was given by a short time, which was evidenced to be protective for intestine against inflammatory damages^26^,^27^. We also found such antibiotics strategy did not exacerbate the inflammation during enteropathy.

Our study design is unique with the use of a FMT model. We showed that the acquired change in microbiota in response to indomethacin protects against NSAID injury. The composition after this adaptive change is significantly different from the original commensals; nevertheless, we also acknowledge that our experiments have not provided mechanistic insights to small bowel enteropathy. In a recent study, antibiotic pretreatment was shown to reduce gut de-glucuronidation and result in increased indomethacin elimination, therefore preventing the mucosae from injury^15^. Antibiotic pretreatment may affect the pathogenesis through altering the drug metabolism. The dynamic bacterial changes in our study may add resilience to the system, and provide an example of colonization resistance against an external insult. Our data provide a strong support for beneficial changes in gut microbiota during the disease development.

Here we demonstrated that indomethacin-induced enteropathy is accompanied with alteration in the gut microbiota and the latter in turn attenuate the indomethacin toxicity. However, identification of the predominant bacterial species which are responsible for the feedback mechanism remains challenging given that many of the gut microbes are difficult to be cultured *in vitro*^28^,^29^. A better understanding of microbiota will be essential for future efforts to target specific subsets of gut microbiota as a therapeutic strategy.

In summary, the study provides new insights on the host-microbiota interactions during NSAID-induced enteropathy. The adaptive changes in gut microbiota provide a negative-feedback mechanism against further injuries.

## Methods

### Animals and experiments design

Animal experiments were approved by the Animal Experimentation Ethics Committee (AEEC) of the Chinese University of Hong Kong. C57BL/6 mice were purchased from the Laboratory Animal Services Centre of the Chinese University of Hong Kong. Mice were bred and housed in the Laboratory Animal Services Centre of the Chinese University of Hong Kong. All experiments were performed in accordance with relevant guidelines and regulations of the Department of Health and the Chinese University of Hong Kong. Adult mice of at least ten weeks of age were used in this experiment.

### Antibiotic depletion model

Depletion of the gut microbiota were performed using an antibiotic cocktail consisting of neomycin (250 mg/kg), polymycin (9 mg/kg) and metronidazole (50 mg/kg) (Sigma Aldrich)^27^.

### Faecal transplant model

Stools were freshly collected from the donor mice daily for 2 days by taking individual mouse out of their cage and collecting the faecal pallet in a micro-centrifuge tube. The stool samples were weighed, diluted by 10-fold volume of sterile saline, vortexed and gravity-settled for 2 minutes. 200 μl stool mixture solution was transplanted to each of the recipient mice by gastric lavage.

### Filtered stool transplantation model

Stool were collected from the donor mice as described above. Slurry stool was filtered with 0.2 m Millipore filter. A total of 200 μl of faecal filtrate was given to recipient mice by gastric lavage for 5 days. Mice were sacrificed after indomethacin administration for 24 hours.

### Gut microbiota and indomethacin-induced enteropathy

Mice were first administered indomethacin (10 mg/kg) for 24 hours, and then received a 5-day course of antibiotics to deplete the gut microbiota. Their body weight, 24-hour faecal weight and survival status were recorded and compared. The same animal experiments were repeated by reducing the dosage of indomethacin to 5 mg/kg and duration of the treatment to 3 days. To study the effects of transplanted microbiota on subsequent indomethacin-induced enteropathy, mice were given a single oral dose of 10 mg/kg indomethacin after 5 days of faecal transplantation and were sacrificed 24 hours afterwards.

### Sample collection and preparation

The presence and extent of intestinal injury were evaluated and recorded. The mice small bowel was sampled in 1 cm segment for every 5 cm of small bowel length. Samples were snap frozen in liquid nitrogen and kept at −80 °C until analyzed. For formalin or Carnoy’s solution fixed sample, 5 mm tissue was taken from every 5 cm small bowel length, and paraffin sectioning was performed for every segment fixed. This gave a total of 4–6 segments for a single mouse. Blood was collected using the retro-orbital approach.

### Stool microbiota analysis by 454 pyrosequencing of 16S rRNA gene amplicons

Total genomic DNA per 100 mg of stool was extracted by QIAamp DNA Stool Mini Kit (QIAGEN) with a bead-bearing method^30^ and quantified by Agilent 2100 Bioanalyzer. PCR amplification of the V1–3 regions of bacterial 16 S rRNA genes was performed using fusion primers comprised of Roche adaptor sequences, Multiplex Identifier (MID) tags, library keys, and template-specific sequences (27F, 5′-GAGTTTGATCMTGGCTCAG-3′; 518R, 5′-WTTACCGCGGCTGCTGG-3′). PCR products were purified by Agencourt AMPure XP system (Beckman Coutler), quantified by Quant-iT PicoGreen dsDNA Assay Kit (Life Technologies), and quality-controlled by removing short amplicon fragments for unidirectional sequencing on the 454 GS FLX Titanium platform according to the manufacturer’s instructions.

### Post-sequencing quality control and sequence read analysis

Raw sff files were processed as described in mothur^31^. Sequences were de-noised using the PyroNoise algorithm. Reads were subsequently demultiplexed, further processed by retaining all that had homopolymers of 8 nucleotide bases or fewer with no ambiguous base call, and aligned against the SILVA database (version 119) using the NAST algorithm. Aligned sequences were screened by optimizing start-, end-coordinates and minimum read lengths, and trimmed to the same alignment coordinates for pre-clustering by a maximum difference of 3 nucleotide bases. Chimeric sequences were detected and culled using de novo UChime. The resulting sequences were annotated against the SILVA database (version 119) using the naïve Bayesian classifier with pseudo-bootstrapped (n = 1,000) confidence scores of at least 80%. Any sequences of eukaryotic, archaic, mitochondrial, chloroplastic, or unknown origins were removed. Finalized sequences were binned into operational taxonomic units (OTUs) by average neighbor clustering and assigned to phylotypes with confidence scores of at least 80. All sequence count tables were subsampled down to the smallest number of reads per sample for differential abundance analyses using the LEfSe bioinformatics package^32^.

### Metagenomic inference

Final sequences were further classified and picked against the Greengenes reference taxonomy and OTU identifiers, respectively (version 13.5). Functional content of the Greengenes-based 97% OTU phylogeny tree was imputed using the PICRUSt algorithm (version 1.0.0)^33^. The predicted metagenomic dataset was summarized by 6,909 KEGG Orthologous (KO) entries and mapped against the metabolic modules of the KEGG BRITE Database (http://www.genome.jp/kegg/brite.html) using the HUMAnN pipeline (version 0.99)^34^.

### Mucosal and faecal bacteria quantification by quantitative PCR (qPCR)

Genomic DNA was extracted from small bowel tissue or stool sample with QIAamp DNA Mini Kit or QIAamp DNA Stool Mini Kit (QIAGEN) separately. For mucosal bacteria quantification, 100 ng total genomic DNA extracted from small intestine (including mouse DNA and bacteria DNA) were used for the following test. Quantitative PCR was performed by using the universal bacteria-specific primer (8F 5′-AGAGTTTGATCCTGGCTCAG-3′, 338R 5′-CTGCTGCCTCCCGTAGGAGT-3′), Bacteriodetes-specific primer (5′-GGTTCTGAGAGGAGGTCCC-3′ and 5′-GCTGCCTCCCGTAGGAGT-3′) and Firmicutes-specific primer (5′-GGAGYATGTGGTTTAATTCGAAGCA-3′ and 5′-AGCTGACGACAACCATGCAC-3′). Standard curves for universal bacteria-specific primer were prepared from serial dilution of *Escherichia coli* genomic DNA. All samples were run in duplicate.

### Clinical assessment of indomethacin-induced injuries

Macroscopic scores were given based on the following criteria modified from Vicente upon laparotomy^35^: A. abdominal distension (0, absent; 1, present), B. presence of adherence (0, no adherence; 1, some local adherence; 2, extensive and generalized adherence), C. appearance of the small intestine (0, normal; 1, moderate signs of congestion and/or distension; 2, clear and generalized alterations), D. appearance of bowel contents (0, normal; 1, reduced content with some fluid or mucus; 2, no content but with fluid or mucus, and blood).

### Quantification of injury area percentage

Quantification of injury lesion in the small bowel was performed on separate mice. Mice were euthanized and small intestines were fixed in 2% formalin solution immediately after removal. Surgical incisions were made along the mesenteric border and the areas of small intestinal injury and total small intestine were quantified with Image J software. Injury area percentages (total ulcer area/total small intestinal mucosae area) were calculated and used for statistic analysis.

### Assessment of histological changes

Histologic changes in H&E-stained paraffin-embedded sections were scored accordingly^10^ (0, normal; 1, mild sloughing of surface epithelial cells; 2, moderate sloughing of surface epithelial cells; 3, extensive mucosal edema or mucosal injury extending deeper than gastric pits; 4, extensive mucosal injury). The pathology scores of slides from one mouse were summed up for statistical analyses.

### Stool haemoglobin concentration examination

Fresh collected stool was diluted by 20-fold with Milli-Q water, vortexed, and briefly centrifuged, the supernatant was used for haemoglobin concentration test with QuantiChromTM Haemoglobin Assay Kit (DIHB-250, BioAssay Systems).

### Real-time PCR

Small intestinal tissues were ground in liquid nitrogen. The total RNA was extracted by TRIzol (Life Technologies) according to the manufacturer’s protocol. cDNA was synthesized using 1 μg of total RNA and amplified with SYBR Green Master Mix (Applied Biosystems) on LightCycler 480 System (Roche). All primers used are shown in Supplementary Table S2.

### Western blotting

Total protein was extracted in radio-immunoprecipitation assay buffer (Thermo). Nuclear protein was extracted with NE-PER Nuclear Protein Extraction Kit separately (Thermo). Denatured proteins were separated on 7.5–12% sodium dodecyl sulfate-polyacrymide gels and transferred to nitrocellulose membranes. The membranes were developed with ECL kit (Amersham Pharmacia Biotechnology, Buckinghamshire, UK) and exposed with X-ray films (Amersham Pharmacia Biotechnologym Buckinghamshire, UK) on ChemiDocTM MP System (Bio-Rad). Intensity of bands was quantified using Image J software. Anti-COX-2 (#12282), anti-phospho-MEK1/2 (#9154), anti-phospho-ERK1/2 (#4370), anti-phosphate-NF-κBp65 (#3033), anti- NF-κBp65 (#8242), and anti-GAPDH (#2118) antibodies were purchased from Cell Signaling Technology; anti-COX-1 (sc-1752), anti-TLR5 (sc-10742), anti-STAT3 (sc-482) and anti-phospho-STAT3 (sc-8001-R) antibodies were purchased from Santa Cruz.

### Enzyme-linked immunoassay (ELISA)

Nuclear protein was extracted by NE-PER Nuclear Protein Extraction Kit (Thermo). NF-κB (p65) binding activity and PGE2 were detected by NF-κB (p65) Transcription Factor Assay Kit (10007889, Cayman chemical), Prostaglandin E2 EIA kit (514010, Cayman chemical), respectively, according to the manufacturers’ instructions.

### Goblet cell quantification

Goblet cells and the nuclei were stained with Alcian Blue solution and Nuclear Fast Red (N8002, Sigma), respectively. Cell count was performed in high power field (×400) (Olympus); three different areas in the same slide were checked and the average was calculated. All slides from one mouse were included in, and the average goblet cell number of all slides was calculated for statistic analysis.

### Statistics analysis

Sequencing data were analyzed as above. The variables of two groups were compared with Student’s *t* test. Survival data were analyzed by Kaplan-Meier survival curve and the log-rank test. Repeated two-way ANOVA was performed to analyze the variation of body weight and faecal weight during days. SPSS statistical software package (standard version 20.0) was used. *P* ≤ 0.05 was noted as significant.

## Additional Information

**How to cite this article:** Xiao, X. *et al*. Gut Microbiota Mediates Protection Against Enteropathy Induced by Indomethacin. *Sci. Rep.* **7**, 40317; doi: 10.1038/srep40317 (2017).

**Publisher's note:** Springer Nature remains neutral with regard to jurisdictional claims in published maps and institutional affiliations.

## Supplementary Material

Supplementary Data
