# Supplementary material for: Gut Microbiota Mediates Protection Against Enteropathy Induced by Indomethacin
Source: Sci Rep. 2017 Jan 9;7:40317. doi: 10.1038/srep40317 (PMC5220306; doi:10.1038/srep40317)
Supplement: Supplementary Data [file srep40317-s1.pdf]

Gut Microbiota Mediates Protection Against Enteropathy Induced by Indomethacin

Xue Xiao[1,2,3], Geicho Nakatsu[2], Ye Jin[2], Sunny Wong[2], Jun Yu[2,*] , James YW Lau[2,3,*]

[1]Department of Gastroenterology, West China Hospital, Sichuan University, Chengdu, China

[2]Institute of Digestive Disease and Department of Medicine and Therapeutics, State Key Laboratory of Digestive Disease, Li Ka Shing Institute of Health Sciences, The Chinese University of Hong Kong, Hong Kong

[3]Department of Surgery, The Chinese University of Hong Kong, Hong Kong

Corresponding authors: Professor James YW Lau, Department of Surgery, Prince of Wales Hospital, The Chinese University of Hong Kong, Shatin, N.T., Hong Kong. Email: laujyw@surgery.cuhk.edu.hk, Phone: (852) 26321411, Fax: (852) 26377974; or Professor Jun Yu, Institute of Digestive Disease, Department of Medicine and Therapeutics, Prince of Wales Hospital, The Chinese University of Hong Kong, Shatin, N.T., Hong Kong. Email: junyu@cuhk.edu.hk, Phone: (852) 37636099, Fax: (852) 21445330.

Supplementary Figure S1:

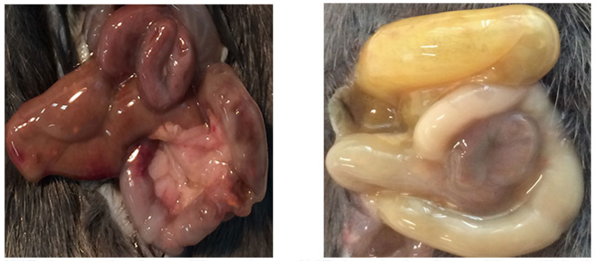

IND treated mice with water   IND treated mice with antibiotics

Supplementary Figure S1: Representative pictures from postmortem examination, showing bleeding lesion in the only dead mouse from IND treated mice with water group, and peritonitis in the dead mice from IND treated mice with antibiotics group.

Supplementary Figure S2:

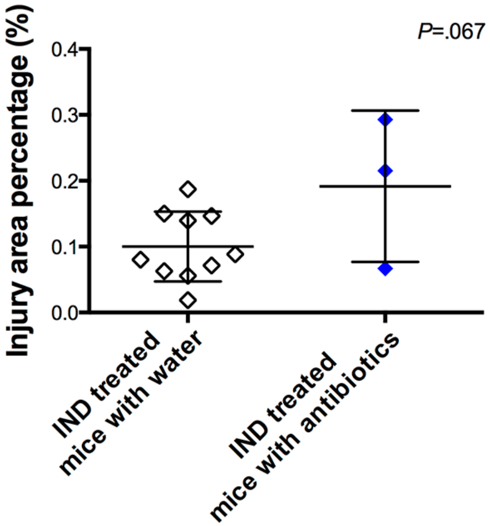

Supplementary Figure S2: Eradication of altered microbiota with antibiotics induced a worse prognosis of indomethacin enteropathy. The survived mice in each group were sacrificed on Day 7 (endpoint, 7 days later after IND treatment), injury area percentage in small bowel mucosa was quantified, every dot represents one mouse.

Supplementary Figure S3:

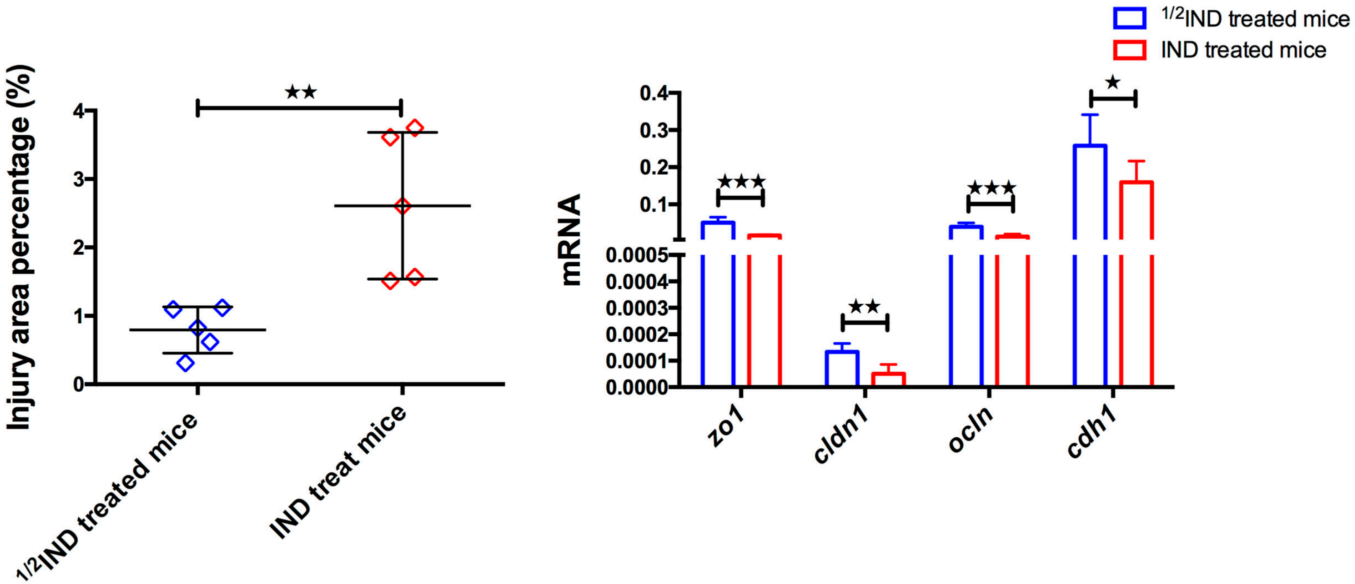

Supplementary Figure S3: Expressions of genes involved in the epithelial tight junctions were reduced according to the severity of indomethacin-induced enteropathy. Two dosage of indomethacin were used to induce different injury extents, as shown in the left graph, mice were sacrificed 24h later to check small bowel injury ($^{1/2}$IND means 5mg/kg indomethacin, IND means 10mg/kg indomethacin). The left graph shows real-time PCR analysis of tight junction genes in the small bowel (*ZO1*, tight junction protein 1; *Cldn1*, claudin 1; *Ocln*, occludin; *Cdh1*, cadherin), n=6 for each group. ★$P \leqslant 0.05$, ★★$P \leqslant 0.01$, ★★★$P \leqslant 0.001$.

Supplementary Figure S4:

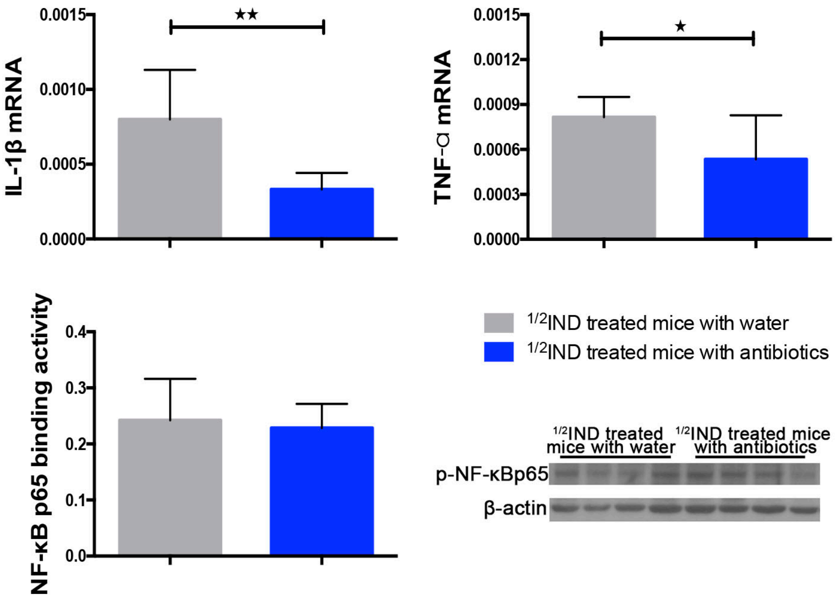

Supplementary Figure S4: Inflammatory cytokines (TNF-α, IL-1β) and NF-κB p65 binding activity were suppressed after antibiotics, nuclear protein was used for western blot. Student's t test was used, ★$P\leqslant0.05$, ★★$P\leqslant0.01$.

Supplementary Figure S5:

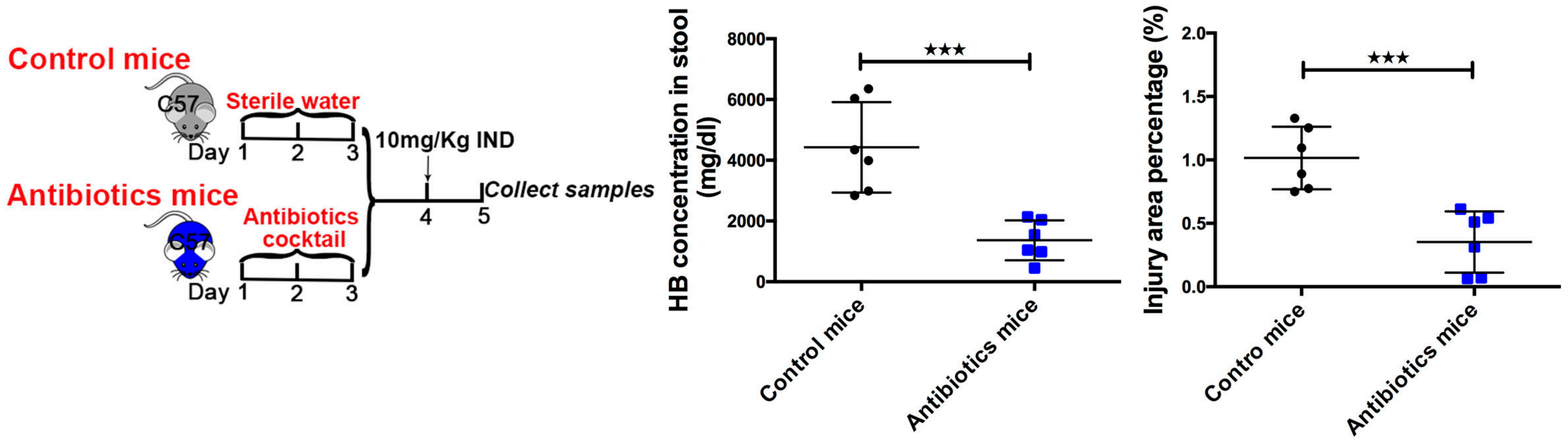

Supplementary Figure S5: Antibiotics pretreatment protected mice from indomethacin induced enteropathy. Experiment scheme, stool haemoglobin concentration data and injury area percentage data are shown, every dot represents one mouse, mean and SD are shown, Student's t test was used, ★★★ $P \leqslant 0.001$.

Supplementary Figure S6:

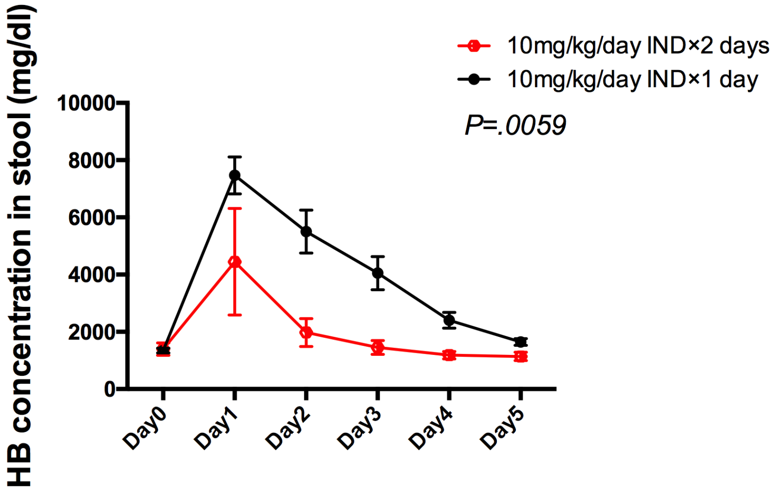

Supplementary Figure S6: Stool haemoglobin concentration in mice treated with different courses of indomethacin. In the Day 0, all of the two groups of mice had not been treated, the Day 1 means 1 day later after mice had been treated with a single dosage of 10 mg/kg indomethacin, or with once daily 10 mg/kg indomethacin treatment for 2 days. n=5-8, according to the Table S1. Repeated two-way ANOVA was used.

Supplementary Table S1: Mice treated with different courses of indomethcian

Treatment		Mice number	Fresh stool[2]		Survival rate[3]
Drug	Days[1]		Yes	No	
10mg/kg/day IND	1	8	8	0	7/8
	2	8	5	3	5/8
	3	8	1	7	1/8

[1]: Mice were treated with IND (indomethacin) once daily for different days.

[2]: Fresh stool were checked 24 hours later after the last IND dose.

[3]: Surivial rate were determined 2 weeks later after finishing the IND administration.

Supplementary Table S2: Mouse primer set for Real-time PCR

Gene	Primer
COX-1	Forward: CGATCTGGCTTCGTGAAC<br>Reverse: GAGCTGCAGGAAATAGCC
IL-1β	Forward: TCAGGCAGGCAGTATCACTCATT<br>Reverse: GGAAGGTCCACGGGAAAGA
TNF-α	Forward: CGTGCTCCTCACCCACAC<br>Reverse: GGGTTCATACCAGGGTTTGA
sucrase-isomaltase	Forward：CAACCTCGGCAAAACCTTTATAGT<br>Reverse: TGCAGCCTCTCTCTACGCAA
Cryptdin	Forward: CAGCCGGAGAAGAGGACCAG<br>Reverse: TAGCATACCAGATCTCTCAACGATTC
defensin	Forward: TCGTTCTGCTGGCCTTCC<br>Reverse: CCTGGCTGTTCCTCAGTTTTAGTC
TLR4	Forward: TGTTGCCCTTCAGTCACAGAGACTCTG<br>Reverse: TGTTGGGTCGTTTGTTCGGATCCGTCG
TLR5	Forward: ATGGATGCTGAGTTCCCCCA<br>Reverse: AAAGGCTATCCTGCCGTCTG
MUC1	Forward: GAGCCAGGACTTCTGGTAGGCT<br>Reverse: GGCTTCACCAGGCTTACGTAGT
MUC2	Forward: TCGCCCAAGTCGACACTCA<br>Reverse: GCAAATAGCCATAGTACAGTTACACAGC
MUC5	Forward: GATCCATCCATCCCATTTCTACC<br>Reverse: TTGCTTATCTGACTACCACTTGTTGA
GAPDH	Forward: AACGACCCCTTCATTGAC<br>Reverse: TCCACGACATACTCAGCA
Ocln	Forward: GATGCAGGTCTGCAGGAGTA<br>Reverse: TCCCACCATCCTCTTGATGT
Cdh1	Forward: CCTGCCAATCCTGATGAAAT<br>Reverse: GTCCTGATCCGACTCAGAGG
ZO1	Forward: CCCTGAAAGAAGCGATTCAG<br>Reverse: CCCGCCTTCTGTATCTGTGT
Cldn1	Forward: TTAGTGGCCACAGCATGGTA<br>Reverse: GAAGGTGTTGGCTTGGGATA
F4/80	Forward: CTTTGGCTATGGGCTTCCAGTC<br>Reverse: GCAAGGAGGACAGAGTTTATCGTG
